# Nanohydroxyapatite/Titanate Nanotube Composites for Bone Tissue Regeneration

**DOI:** 10.3390/jfb13040306

**Published:** 2022-12-17

**Authors:** Suziete B. S. Gusmão, Anupama Ghosh, Alan S. de Menezes, Antônio F. M. Pereira, Miriam T. P. Lopes, Madaline K. Souza, Dalton Dittz, Guilherme J. P. Abreu, Lucielma S. S. Pinto, Antônio L. M. Maia Filho, Gustavo O. M. Gusmão, Thomas J. Webster, Anderson O. Lobo, Bartolomeu C. Viana

**Affiliations:** 1 Interdisciplinary Laboratory for Advanced Materials(LIMAV), Materials Science and Engineering Graduate Program, Federal University of Piaui, Teresina 64049-550, PI, Brazil; 2 Central Analítica, Federal University of Ceara, Fortaleza 60020-181, CE, Brazil; 3 Department of Chemical and Materials Engineering—DEQM, Pontifical Catholic University of Rio de Janeiro, Rio de Janeiro 22541-041, RJ, Brazil; 4 Departamento de Física, CCET, Federal University of Maranhao, São Luís 65080-805, MA, Brazil; 5 Departamento de Enfermagem, Federal University of Piaui, Teresina 64049-550, PI, Brazil; 6 Departamento de Farmacologia, Federal University of Minas Gerais, Belo Horizonte 31270-901, MG, Brazil; 7 Departamento de Bioquímica e Farmacologia, Federal University of Piaui, Teresina 64049-550, PI, Brazil; 8 Departamento de Física, Universidade Federal do Paraná, Curitiba 80060-000, PR, Brazil; 9 Centro de Ciências da Saúde, State University of Piaui, Teresina 64049-550, PI, Brazil; 10 Núcleo de Pesquisa em Biotecnologia e Biodiversidade, State University of Piaui, Teresina 64002-150, PI, Brazil; 11 Departmento de Física, State University of Piaui, Teresina 64049-550, PI, Brazil; 12 School of Health Sciences and Biomedical Engineering, Hebei University of Technology, Tianjin 300130, China; 13 School of Engineering, Saveetha University, Chennai 602105, India

**Keywords:** nanocomposite, titanate nanotube, hydroxyapatite, bone repair

## Abstract

Strategies for the production of new nanocomposites that promote bone tissue regeneration are important, particularly those that enhance the osteoinduction of hydroxyapatite in situ. Here, we studied and report the synthesis of nanohydroxyapatite and titanate nanotube (nHAp/TiNT) composites formulated at different concentrations (1, 2, 3, and 10 wt % TiNT) by means of a wet aqueous chemical reaction. The addition of TiNT affects the morphology of the nanocomposites, decreasing the average crystallite size from 54 nm (nHAp) to 34 nm (nHAp/TiNT10%), while confirming its interaction with the nanocomposite. The crystallinity index (CI) calculated by Raman spectroscopy and XRD showed that the values decreased according to the increase in TiNT concentration, which confirmed their addition to the structure of the nanocomposite. SEM images showed the presence of TiNTs in the nanocomposite. We further verified the potential cytotoxicity of murine fibroblast cell line L929, revealing that there was no remarkable cell death at any of the concentrations tested. In vivo regenerative activity was performed using oophorectomized animal (rat) models organized into seven groups containing five animals each over two experimental periods (15 and 30 days), with bone regeneration occurring in all groups tested within 30 days; however, the nHAp/TiNT10% group showed statistically greater tissue repair, compared to the untreated control group. Thus, the results of this study demonstrate that the presently formulated nHAp/TiNT nanocomposites are promising for numerous improved bone tissue regeneration applications.

## 1. Introduction

Most of the time, bone replacements and artificial implants are necessary for treating orthopedic problems, such as accidental injuries caused by old age, trauma from traffic accidents, degenerative diseases (osteoporosis), etc. [1,2]. Thus, bone substitute/repair materials are increasingly replacing the use of bone grafts and have been adopted as a primary bone tissue engineering strategy. Bioactive materials and/or different classes of functional materials (such as metallic, ceramic, and polymeric), which can be used alone or in combination as composites, have become key materials of interest. Regardless of their composition, once implanted in bone, promising bone replacement materials mimic the bone extracellular matrix, inducing a favorable cell-implant response [3].

HAp is a biomaterial extensively used as a bone graft in orthopedic surgeries, having a chemical composition (Ca_10_(PO_4_)_6_(OH)_2_) that is bioactive, biocompatible, biodegradable, osteoconductive, and non-toxic [4]. However, the fragile behavior of HAp, particularly its low wear resistance, has kept it from widespread clinical use, especially under mechanical loading conditions [5,6,7].

In this regard, the possibility of developing new and improved materials with special properties designed for implants to replace or repair hard tissues has encouraged researchers around the world to develop nanocomposites based on nanobiomaterials that can mimic the fundamental nano building blocks of natural, healthy bone. Nanobiomaterials reduce the risk of rejection by the human body, as they are biocompatible and biofunctional; present a high resistance to degradation from physiological body fluids; and have high fracture toughness and hardness, all factors considered important in the design of improved bone implants. In addition, the biomimetic and high surface energetics of nanobiomaterials provide a fast and predictable response to biological tissue and body fluids [8].

One of the most common approaches to overcome such weaknesses and provide better wear resistance includes incorporating reinforcing materials such as titanium, which is preferred and used in orthopedic prostheses because it has an elastic modulus closer to that of bone [9,10]. However, the longevity of titanium as a bone implant sometimes fails after prolonged use due to an incomplete union between the implant and surrounding bone [11]. In order to improve material interaction and increase longevity, bioactivity, and bone growth, surface treatments such as roughening, hydroxyapatite coating, formation of titanate phases, or chemical treatments [12,13] have been used.

Based on this, HAp-based nanocomposites have been extensively investigated in order to improve their mechanical properties, combining their composition with other bioactive nanomaterials, such as carbon nanotubes (CNTs) or nanotubes of TiO_2_ or ZrO_2_ [11,12,13].

Nanotubes based on the titanium oxide structure, such as TiNT, are some of the most promising inorganic nanomaterials for orthopedics, in that they have a high surface area and ion exchange capacity and the possibility of surface modification for biomedical applications. Ti is the most implantable orthopedic biomaterial today, and TiNTs can be easily applied as biomimetic and bactericide materials [14,15]. TiNTs have tubular shapes and diameters below 10 nm with a greater capacity for internalization into cells, compared to spherical particles [7], opening up numerous bone tissue engineering applications.

Herein, we report the synthesis of nanocomposites composed of nHAp/TiNTs at different weight concentrations of TiNTs (1%, 2%, 3%, and 10%) for bone regeneration applications. Nanocomposites not only are not cytotoxic but also even showed greater bone tissue regeneration after 30 days during in vivo implantation (specifically, the nHAp/TiNT10% group promoted bone growth the most, compared to the control group without any treatment). As such, this study introduces a novel new orthopedic implant composed of nHAp and TiNTs, as proven by promising in vivo studies.

## 2. Materials and Methods

### 2.1. Synthesis of the TiNTs

The TiNTs were synthesized by a conventional hydrothermal method based on the synthesis reported in Kasuga et al. [16], which was performed using the procedure adapted from Wang et al. [17] and Marques et al. [18]. In this process, using an alkaline hydrothermal method, 2.00 g of TiO_2_-anatase powder (Sigma-Aldrich, free from other metal ions, average particle size around 60–80 nm, 99.8% purity) was dispersed in 60 mL of an aqueous solution of sodium hydroxide (NaOH, Sigma-Aldrich, 97.0% purity) of concentration 10 mol L^−1^ and magnetically stirred for 30 min at room temperature. The white suspension formed was transferred to a Teflon-lined stainless-steel autoclave, taken to a greenhouse oven, and hydrothermally reacted at 140 °C for 96 h. Then, it was cooled down to room temperature, when the materials were centrifuged at 3500 rpm for 5 min and washed (with deionized water) several times to remove the excess sodium ions and other impurities from the surface of the material until the final pH reached 9. Subsequently, the products were dried in a vacuum at room temperature for 48 h.

### 2.2. Preparation of a nHAp/TiNT Nanocomposite

The nHAp/TiNT nanocomposites were prepared at different concentrations of TiNTs (1, 2 and 3 wt%). The solution used for all of the samples were as follows: 100 mL of a 0.167 M calcium nitrate tetrahydrate (Ca(NO_3_)_2_·4H_2_O) solution (Sigma-Aldrich, 99.0% purity) and 100 mL of a 0.1 M of diammonium hydrogen phosphate ((NH_4_)_2_HPO_4_) solution (Sigma-Aldrich, 98.0% purity). TiNTs were added under sonication (Vibracell Sonics, 500 W, 20 kHz, 13 mm probe, model: SO-VCX-500, SONICS) for 30 min; these initial proportions yielded a theoretical calcium to phosphorous ratio (Ca/P) of 1.67. The pH of both solutions was fixed at ≈ 10, and, every 10 min, the dropwise addition of an ammonium hydroxide solution (NH_4_OH-25%) was performed. The precipitate was left to rest for 120 h, corresponding to the maturation time, then was centrifuged at 3500 rpm for 5 min, washed several times with isopropyl alcohol and deionized water, and oven-dried for 48 h at 80 °C. After drying, the samples were denominated as nHAp/TiNT at 1, 2, 3, and 10 wt % [19].

### 2.3. Characterization of nHAp, TiNTs, and nHAp/TiNTs

#### 2.3.1. Raman Spectroscopy

Raman spectroscopy was performed at room temperature in a Raman spectrometer, Bruker Senterra II Micro-spectrometer, using 10X objective lenses. A laser at an excitation of 785 nm and a power output of 50 mW was used. The spectrometer grating was adjusted to obtain a spectral resolution of 3 cm^−1^ sweeping in a range of 85–1530 cm^−1^. The crystallinity index (CI) was also calculated using Raman spectroscopy and obtained through a profile of the highest intensity band, 961 cm^−1^, which was assigned to the symmetrical stretching vibration of the ${\mathrm{PO}}_{4}^{3-}$ group of phosphate. The FWHM values were collected after deconvolution of this band using standard and Lorentz-type profiles. The CI was estimated by using Equation (1).

(1) $${\mathrm{CI}}_{\mathrm{Raman}}=\frac{4.9\;}{\Gamma }\;\;$$

where the value of 4.9 refers to the average FWHM of the magmatic apatite standard [20] with high crystallinity, and Γ is the FWHM of the peak at 961 cm^−1^.

#### 2.3.2. X-ray Diffraction

The X-ray diffraction (XRD) patterns were obtained using a Bruker model D8 advance diffractometer, equipped with a linear lynxeye detector, using Cu-Kα radiation (λ = 1, 5406 Å), with a tube operating at 40 kV and 40 mA. Data were collected in a 2θ range from 5° to 70°, with a 0.02° step size and a 0.5 s/step.

The diffractograms obtained were compared with the nHAp crystallographic file at the International Diffraction Data Center (JCPDS) No. 01-074-0566. For Rietveld refinement analysis, we used the diffraction patterns from the pure HAp Inorganic Crystal Structures Database (ICSD) No. 161328, and these were used as an initial model of structural refinement [21]. Thus, Rietveld Refinement was performed using the GSAS [22] program to obtain the lattice parameters, unit cell volume, anisotropic crystallite size, and microstrain.

The crystallinity index was estimated through the planes (hkl) by X-ray diffraction and was calculated using the following empirical relationship (Equation (2)) [23].

CIx-ray = 1 − (V_112/300_/I_300_) (2)

where CIX-ray is the crystallinity, I300 is the intensity of the reflection (300), and V_112/300_ is the intensity of the void between the reflections (112) and (300), which disappears completely in samples that are not crystalline. Based on the Debye–Scherrer equation (Equation (3)), we can estimate the average size of the nanoparticles [19].

(3) $$\tau =\frac{k\lambda }{\beta \cos\theta }\;$$

where τ is the size of the crystallite in nanometers, k is the constant considered as a spherical shape (in this case, k = 0.9), λ is the wavelength of CuKα radiation (1.54 Å), β is full width at half-maximum (FWHM) of the (002) peak, and θ is the diffraction angle (°) satisfying Bragg’s law.

#### 2.3.3. Attenuated Total Reflectance Fourier-Transform Infrared Spectroscopy (ATR–FTIR)

Fourier-transform infrared (FTIR) spectroscopy was recorded using an attenuated total reflectance (ATR) accessory on a Vertex 70 (Bruker, Germany) spectrometer, employing a Ge crystal. A total of 64 scans and a resolution of 4 cm^−1^ were used to obtain spectra with good signal-to-noise ratios. The range used was 4000–600 cm^−1^, and the measurements were taken at room temperature.

#### 2.3.4. Particle Size Analysis and Zeta Potential

The average particle size and the polydispersity index (PDI) of the samples under study were determined by dynamic light scattering (DLS) using Zetasizer Nano ZS equipment from Malvern Instruments (model DTS 1070, Malvern, UK) with a polycarbonate cuvette equipped with beryllium/copper-coated gold electrodes. The sample preparation involved the dilution of the nanostructures in an aliquot of 1 mL of ultrapure water, dispersed in an ultrasonic bath for 20 min, and then the analysis was performed at an incident angle of 90 °C to 25 °C. Additionally, the samples were placed in capillary tubes and analyzed at 25 °C to estimate the surface charge values by Zeta potential.

#### 2.3.5. Scanning and Transmission Electron Microscopy

SEM images were obtained using a Quanta 450 FEG microscope manufactured by FEI (Eindhoven, The Netherlands). For SEM analysis, the samples were pulverized on carbon tape attached to aluminum sample holders and coated with gold by sputtering using an ES Quorum QT150. For elemental mapping, energy-dispersive X-ray spectroscopy (EDS) was recorded using an X-ray detector model 150 from Oxford attached to a Quanta 450 FEG microscope (JEOL JEM 1200EX-II Transmission Electron Microscope, Akishima, Japan) with 0.5 nm resolution that allowed for magnifications up to 600 kX. The images were recorded through a CCD Gatan camera (Bioscan, Gatan Inc., Pleasanton, CA, USA) and a high-resolution CCD Gatan camera (Orius SC1000B, Gatan Inc., Pleasanton, CA, USA).

### 2.4. In Vitro Study

#### Cell Viability

The viability of L929 cells (murine fibroblast) after TiNT, nHAp, nHAP/TiNT1%, nHAp/TiNT2%, nHAP/TiNT3%, and nHAP/TiNT10% exposure was evaluated by the resazurin assay [24] and in accordance with ISO 10993-5 [25]. Briefly, exponentially growing cells, seeded in 96-well plates (10^4^ cells/well), were incubated for 24 h with concentrations up to 300 μg/mL of TiNT, nHAp, nHAP/TiNT1%, nHAp/TiNT2%, nHAP/TiNT3%, and nHAP/TiNT10%, which were resuspended in RPMI media supplemented with 5% fetal bovine serum (Sigma Chemical Co, St. Louis, MO, USA). At the end of this period, the medium was removed, and the cells were washed twice with PBS pH 7.4. Then, a new medium containing 10 μL of 10 mg/mL resazurin (Sigma Chemical Co, St. Louis, MO, USA) was added into each well and incubated at 37 °C for 4 h. The absorbance of the medium was determined at 570 and 600 nm using a Multiskan GO ELISA reader (Thermo Fisher Scientific, Fremont, CA, USA), and the graph was plotted as % of viability (mean ± mean standard error), compared to untreated cells (control—Ctr).

### 2.5. In Vivo Study

#### 2.5.1. Ethical Aspect

The experiments described below were carried out in accordance with the guiding principles for the care and use of laboratory animals previously approved by the Ethics Commission on the Use of Animals of the Uninovafapi University Center (CEUA-UNINOVAFAPI), under approval number nº 002P-V2/2019, in accordance with federal law No. 11.794 and with the rules issued by the National Council for the Control of Animal Experimentation.

##### Animals

Seventy eight-week-old female rats (Rattus norgicus albinus Wistar) were used, with average body weights varying between 250 and 300 g, provided by the vivarium of the State University of Piaui. All animals were housed in steel cages with five animals in each, and were fed ad libitum food and water, with rice straw bedding changed every two days, at a temperature of 24 °C, with an interval of 12 h light and 12 h dark.

##### Surgical Procedure

The experimental procedure was developed at the Biotechnology and Biodiversity Center of the State University of Piaui. The samples were divided into six groups: control group of samples (HAp and TiNT) and groups in different concentrations by mass of TiNT (HAp/TiNT1%, HAp/TiNT2%, HAp/TiNT3%, and HAp/TiNT10%). Another group included animals without any treatment (control). In turn, each group was subdivided into two observation periods (a euthanasia period at 15 and 30 days) for all groups, with 5 animals each.

All animals were oophorectomized under anesthesia by the association of ketamine and xylazine intramuscularly and through a subcutaneous incision in the lateral skin approximately 1.5 cm in length below the last rib, for removal of the ovaries, and then the lesions were sutured using 4-0 silk thread. Subsequently, the animals were returned to the cages under the same conditions mentioned above, for a period of 45 days, the time necessary for the onset of osteopenia [20].

**Figure 1:**
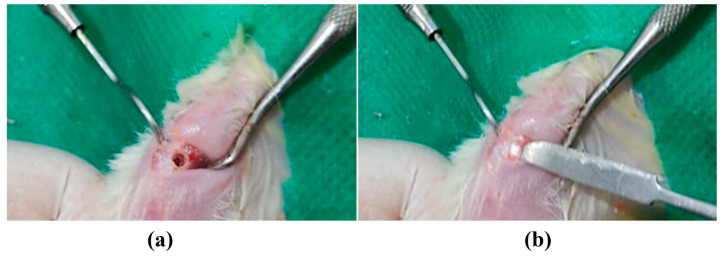
Photographs of the surgical procedure for (**a**) making the bone cavity and (**b**) implantation of the nanobiomaterials.

For the implantation of the nanobiomaterials, the animals were dissociatively anesthetized intramuscularly with a dose of 0.1/0.1 mL of ketamine and xylazine for every 100 g of body weight. After anesthesia, each animal was submitted to epilation in the region of the right tibia with access to the bone tissue performed via an incision on the medial side of the same tibia, and, with the aid of a scalpel blade, the bone tissue was exposed. A critical defect in a circular shape was caused by means of a micromotor, a 3 mm diameter trephine drill (Figure 1a), and, when the surgical cavity was completed, it was ready to receive the proposed treatments (implantation of nanocomposites) (Figure 1b). The control group consisted of the bone cavity, which received no treatment. Soon thereafter, the margins of the lesions were sutured using 4-0 silk thread. After the surgical procedure, the animals received 50 mg/kg of dipyrone by gavage every 12 h as analgesia.

#### 2.5.2. Experimental Period

After 15 and 30 days of implantation of the nanobiomaterial in the animals’ tibia, the animals were euthanized by an anesthetic overdose of sodium thionembutal (150 mg/kg). The tibias of all animals from all groups were removed, preserved in liquid nitrogen, and subsequently submitted to analysis by Raman spectroscopy to verify the bone composition formed. After Raman analyses, the bone tissue samples were stored in formalin for conservation, and, later, they were embedded in paraffin and histopathologically analyzed.

#### 2.5.3. Raman Analysis of Osteogenesis

Bone tissue samples were removed from liquid nitrogen and kept at room temperature. The analysis took place as follows: in the bone region of interest, three spectra were obtained with an approximate distance of 10 μm between the points. A spectrum of the region distant from the lesion was also obtained, as healthy cortical bone. Thus, the Raman spectra collected were classified as healthy (bone), control (without any treatment), and treated with nHAp, TiNT, nHAp/TiNT1%, nHAp/TiNT2%, nHAp/TiNT3%, and nHAp/TiNT10%; these were compared between the treated region and the healthy region. Peaks of interest were identified at between approximately 957 cm^−1^ and 961 cm^−1^ as characteristic of the phosphate group, in order to quantify the bone composition formed as well as to characterize bone alterations and the mineral content; the ratio of the integrated area of the characteristic band of the phosphate group ≈ 960 cm^−1^ was estimated [26,27]. In addition, pre-processing steps, such as fluorescence removal from the sample, were performed, and a fourth-order polynomial was used for each spectrum in the regions of 900–3500 cm^−1^ to infer changes in the mineral component of the bone. A baseline adjustment was performed. Subsequently, the processing was completed performing vector normalization.

#### 2.5.4. Histopathological Analysis

Histological sections were obtained through dissection of bone tissue samples (tibias), which were decalcified in 20% hydrochloric acid for seven days and subsequently dehydrated in alcohol solutions in gradual and increasing concentrations. Then, the samples were treated with xylene and embedded in paraffin, with the long axis of the tibia parallel to the surface of the paraffin block using an automatic tissue processor (PT05 TS Luptec, São Paulo, Brazil). After being embedded in paraffin, sections with a distance and thickness between 2 and 3 μm were performed on a rotary microtome (MRP09 Luptec, São Paulo, Brazil) and stained with hematoxylin and eosin (H&E), two sections per slide.

Histological analyses of bone repair processes were performed by a certified histologist, who was blinded to the treatments performed. Sections of all samples were analyzed under a trinolucular light microscope (Olympus CX31, Tokyo, Japan) and photographed in 10x objective lenses with a digital camera (Moticam WiFi X, MoticMicroscopes, Richmond, VA, USA) attached to a computer. The slides were analyzed, and bone neoformation parameters were adopted for each period considered in the experiment. The semi-quantitative histopathological analysis of tissue repair was performed using a numerical scoring system (scores) adapted from previous work [26,28] The definition of the scores was classified as follows: Score 0—Complete absence of bone repair; Score 1—Incipient bone neoformation restricted to the edges of the lesion; Score 2—More evident bone neoformation with trabeculae composed of immature bone tissue from the edges of the lesion; Score 3—Bone neoformation from the edges and inside the bone defect, but in restricted areas; Score 4—Bone neoformation on the edges and inside the defect, filling almost the entire extension of the bone defect; Score 5—Bone neoformation on the edges and inside the bone defect, filling its entire length, with a thickness of up to 1/3 when compared to the intact bone margins; Score 6—Bone neoformation on the edges and inside the bone defect, filling its entire length, with a thickness greater than 1/3 and less than 2/3 when compared to intact bone margins; Score 7—Bone neoformation on the edges and inside the bone defect, filling its entire extension, with a thickness greater than 2/3 but not yet with a thickness similar to the intact margins; and Score 8—Fully filled bone defect with a thickness similar to the intact edges.

#### 2.5.5. Statistical Analysis

Statistical data were analyzed by analysis of variance, ANOVA, and by the non-parametric Kruskall–Wallis test, since the number of samples in each group was reduced, followed by a Dunn’s post-test. For these analyses, the Graphpad Prism program (version 8.3.0, San Diego, CA, USA) was used. Significant differences were considered when the *p* value was less than 0.005. The results are expressed as their means and standard deviation.

## 3. Results and Discussion

**Figure 2:**
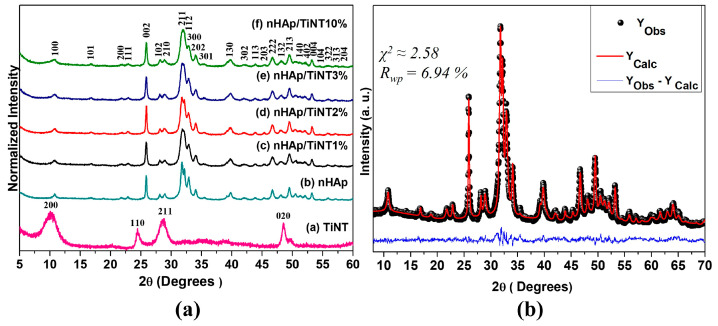
(**a**) X-ray diffractograms for nHAp, TiNT, nHAp/TiNT1%, nHAp/TiNT2%, nHAp/TiNT3%, and nHAp/TiNT10% showing the planes of hydroxyapatite and TiNT and (**b**) Rietveld refinement profile with the observed (black dots), the calculated (red), and the difference (blue) between the observed and calculated data for nHAp sample.

Figure 2a shows the results from the X-ray diffraction analysis of nHAp as synthesized and with different concentrations of TiNTs. To confirm the crystalline phase of the material, refinement was performed by the Rietveld method (Figure 2b). The main characteristics of the hexagonal HAp phase are related to the peaks ≈ 25.8°, 31.8°, 32.2°, 32.9°, and 34.0°, which are related to the crystalline planes (002), (211), (112), (300), and (202), respectively, and other peaks that are indexed with the crystallographic file (JCPDS: 01-074-0566) for HAp. For the TiNT sample, the structure was confirmed by the main characteristic peaks at 2θ ≈ 10.1°, 24.5°, 28.6°, and 48.5°, which corresponded to the crystalline planes (200), (110), (211), and (020), respectively [29]. The XRD results also revealed the formation of materials with higher crystallinity that can be attributed to nHAp in a higher proportion than TiNTs for the formation of the nanocomposites proposed in this work (Figure 2a).

Figure 2b shows the observed and calculated XRD profile of the HAp, as well as the difference between them (Yobs—Ycal), after refinement by the Rietveld method using ICSD nº 161328. The calculated patterns were adjusted to the observed experimental pattern, providing the structural parameters of the desired material and its diffraction profile. The HAp refinement profile shows that there is a good correlation between the observed and calculated intensities and the diffraction patterns (Figure 2b, nHAp/TiNT nanocomposites (1%, 2%, 3%, and 10%)) at different concentrations. Furthermore, XRD profiles did not show any additional phases other than those mentioned in the nanomaterials.

Table 1 shows the nHAp lattice parameters obtained by the Rietveld refinement. Generally, refinement is considered satisfactory when reliability coefficient values such as chi-squared (χ^2^) or goodness of fit (S), empirically obtained by S = Rwp/Rexp, show values S < 2, according to Vieira et al. 2013 [30]. The obtained χ^2^ = 2.58 (S = 1.6) showed good refinement. Rwp values < 10% are considered acceptable for the hexagonal structure of HAp. Thus, the Rwp obtained for our nHAp sample was 6.94%, which was considered a good refinement, when compared to the literature [31,32].

The structure of HAp consists of a hexagonal unit cell with a space group of P63/m. It has 10 calcium ions located in two independent crystallographic sites for calcium (Ca1 and Ca2): four atoms of Ca1 located at the ends of the unit cell and six Ca^2+^ that form triangles parallel to the c axis, located in the corners of the hexagon around OH^−^. The hexagonal unit cell of HAp also has four oxygens (O1, O2, O3, and O4), and the P1 sites are all equivalent, type ${\mathrm{PO}}_{4}^{3-}$, which are bonded with oxygen (O1, O2, and O3) [33]. The lattice parameters, a = b ≠ c and α = β ≠ γ = 120°, as well as their atomic coordinates, confirm the XRD results as a single phase of the HAp structure. In addition, the nHAp crystal is anisotropic, that is, it has two crystallite sizes in relation to its c axis. Rietveld refinement provided a parallel size with a value of 59.2 nm, a perpendicular size of 19.2 nm, and a microstrain value of 0.11%.

**Figure 3:**
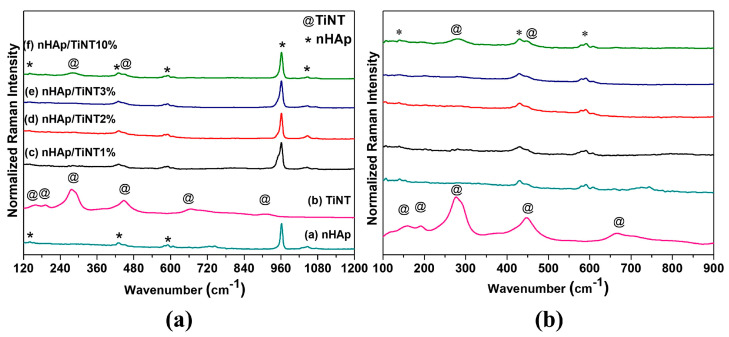
(**a**) Raman spectra for nHAp, TiNT, nHAp/TiNT1%, nHAp/TiNT2%, nHAp/TiNT3%, and nHAp/TiNT10% and (**b**) zoom of the region 100 to 900 cm^−1^.

The Raman spectra are shown in Figure 3. The vibrational modes at 159 cm^−1^ and 192 cm^−1^ are attributed to the lattice vibrations (Na-O-Ti), with 277 cm^−1^, 447 cm^−1^, 666 cm^−1^, and 706 cm^−1^ as the vibrations between the Ti-O-Ti bonds from the TiO_6_ octahedra, and the mode at 908 cm^−1^ is associated with the terminal bonds that are not shared with Ti-O, confirming the titanate nanotube features [29,34]. The nHAp Raman spectrum has a characteristic stretching mode of (${\mathrm{PO}}_{4}^{3-}$) ν_1_ ≈ 961 cm^−1^, as can be seen in Figure 3a, which is the predominant form of crystallized HAp, as well as bands of lower intensities that were also observed and are attributed to vibrations from other apatite groups; modes of ν_2_ (PO_4_) ≈ 431 cm^−1^ octacalcium phosphate bending, ν_3_ (PO_4_) ≈ 587 cm^−1^ dehydrated calcium phosphate, and ν_4_ (PO_4_) ≈ apatite phosphate group bending [20,35] were observed in nHAp and nHAp/TiNT samples at different concentrations (1%, 2%, 3%, and 10%). However, the characteristic bands of TiNTs were observed only in the samples with a concentration of 10% by mass, for TiNTs in the formation of the nanocomposite (Figure 3b).

Based on the results of the crystallinity indices shown in Table 2, it can be seen that the values referring to the crystallinity of the samples decreased with an increase in TiNT mass using both techniques.

Table 3 shows the main reflection peak characteristic of HAp around 25.9°, where the crystallite sizes were calculated using the Scherrer equation based on the width at half height of the reflection (002), as explained in the experimental section. The crystallite size found as 54 nm for pure nHAp tended to decrease with increasing TiNT concentrations, after the formation of nanocomposites, showing a smaller crystallite size in the nHAp/TiNT10% sample, with a value of 34 nm.

**Figure 4:**
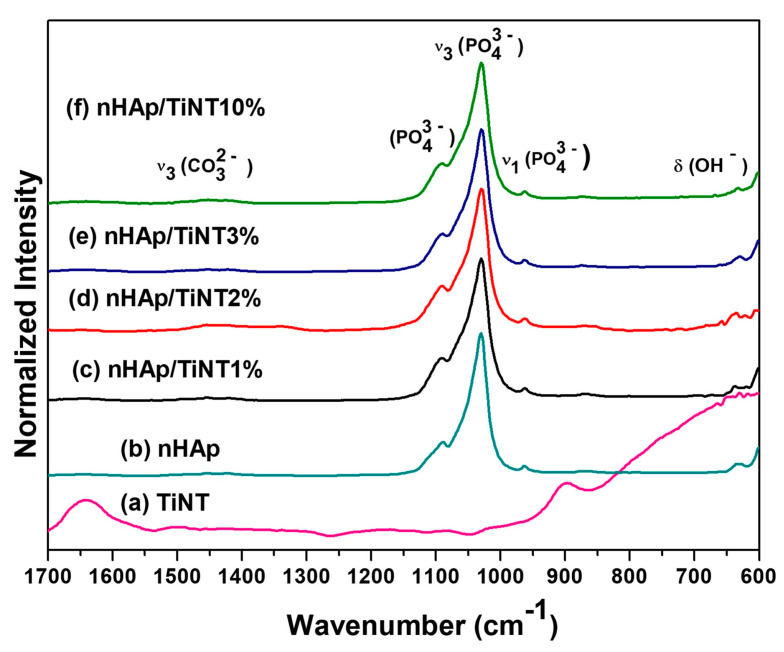
ATR–FTIR spectra of the samples cited in the graph, showing the characteristic peaks of hydroxyapatite and titanate nanotubes.

As shown in Figure 4, the ATR–FTIR spectra were similar among the nanocomposite samples (Figure 4c–f). For the spectrum for the nHAp and nHAp/TiNT samples (1%, 2%, 3%, and 10%), the vibrational modes ≈ 1.029 cm^−1^ and 1.092 cm^−1^ are related to the anionic group (${\mathrm{PO}}_{4}^{3-}$) and are assigned to the triple-degenerate antisymmetric P-O symmetric stretching modes (ν_3_) and mode ≈ 962 cm^−1^, which is the non-degenerate P-O symmetric stretching mode (ν_1_). The band observed at 632 cm^−1^ is attributed to the deformation vibration of the OH^−^ groups of HAp. In addition, a mode at 1.451 cm^−1^ was observed, which is characteristic of the carbonate group (${\mathrm{CO}}_{3}^{2-}$) in the nanocomposites [20,36], showing the presence of calcium carbonate (Figure 4 b–f).

The TiNTs vibrational mode at ≈ 1.643 cm^−1^ can be attributed to the H-O-H bending vibrations of the water molecules. We also observed a band around 899 cm^−1^ that corresponds to the Ti-O bonds involving stretching modes, which are a type of terminal bond coming out from the walls of TiNTs and can form bonds with other molecules (Figure 4a) [37].

The surface properties of nanoparticles are driven, among other factors, by their surface charge. These properties can define their interactions with different biological components, and can also predict the behavior of nanoparticles, through their surface charge (Zeta potential) in biological fluids [38,39]. In Table 4, the Zeta potential measurements and the mean size distribution of the samples characterized using dynamic light scattering (DLS) are listed.

The Zeta potential analysis of the nanocomposites revealed that they have a low negative Zeta potential, varying in surface charges from −0.019 mV to −1.520 mV, as shown in Table 4. The values for the TiNT and nHAp samples ranged from −27.40 mV to −5.16 mV, respectively. The negative values of Zeta potential can be attributed to the presence of functional groups such as the hydroxyl groups on the surface of the materials, as evidenced by the FTIR spectra (Figure 4). The high negative Zeta potential value for the TiNTs can be attributed to the large number of these chemical groups on their surface.

Hydrodynamic diameters were between 2247 nm to 3824 nm (Table 4). These high diameters may be associated with possible agglomerations of these nanoparticles due to intense surface loading, as indicated by the Zeta potential measurements.

**Figure 5:**
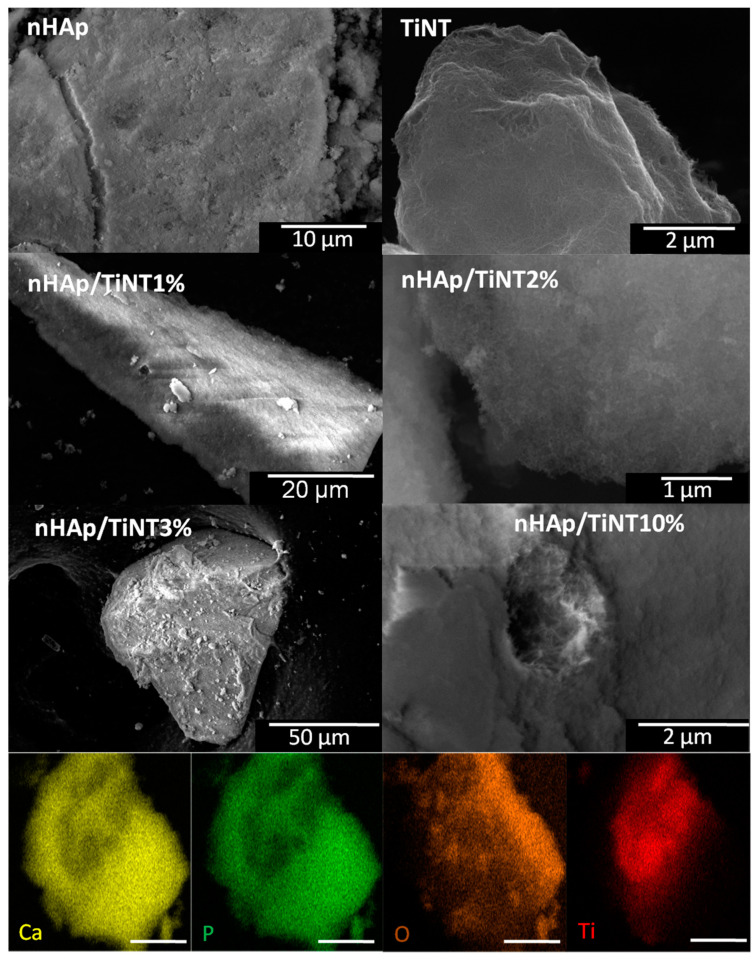
Scanning electron microscopy images of nHAp, titanate nanotubes, and nHAp/TiNT nanocomposites at different concentrations (1, 2, 3, and 10% wt) of TiNT and elemental mapping of nHAp/TiNT10%.

The morphological analysis observed through the SEM (Figure 5) and TEM images (Figure S1) showed that all samples were very small particles in agglomerate, forming plates. The presence of titanate nanotubes was also observed for nHAp/TiNT10%. Furthermore, in Figure S2, the nanometric behavior of the titanate nanotubes can be observed. Elementary mapping via EDS was carried out to determine the chemical composition of the surface of the samples, showing the formation of nanocomposites with TiNT concentrations between 1%, 2%, and 3% and the presence of Ti in small amounts, as can be seen in Table 5. However, as expected, at a concentration of 10% TiNT, the presence of Ti was much more considerable (Figure 5).

Through the EDS analysis, it was possible to calculate the Ca/P atomic ratio, as shown in Table 5. It was found that the Ca/P atomic ratio was 1.93 for nHAp and that there was an increase in the sample at a concentration of 3% of TiNT, with the highest value of Ca/P being 3.03 for nHAp/TiNT3%. However, according to these results for the Ca/P ratios obtained through semiquantitative EDS analyses, we cannot suggest that the type of HAp synthesized in this work is the type that presents typical values of Ca/P between 1.50 and 1.67, as in previous work; therefore, further characterization would be necessary [32]. For example, this higher ratio of calcium may be related to the formation of calcium carbonate on the surface of the nanomaterials.

**Figure 6:**
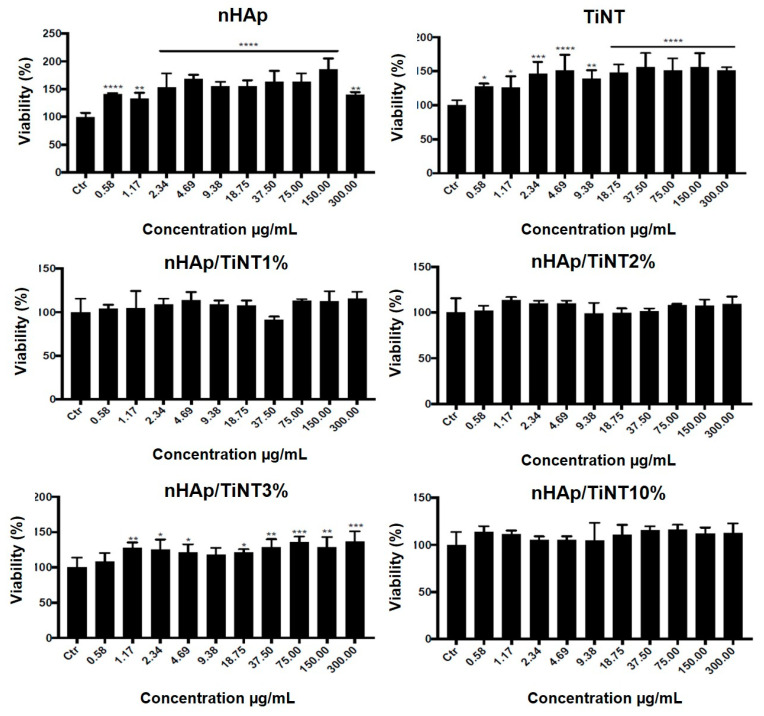
The cytotoxicity analyses of nHAp/TiNT nanocomposites at different concentrations (1%, 2%, 3%, and 10% wt of TiNT) on L929 cells. Cell viability was determined after 24 h of exposure of the nanocomposites at concentrations up to 300 μg/mL. Data are expressed as ± standard error; * *p* < 0.05, ** *p* < 0.01, *** *p* < 0.001, and **** *p* < 0.0001, compared to controls; unidirectional ANOVA (Dunnett post-tests).

Considering the importance of developing a biocompatible material, the cytotoxic activity of all the samples in this study were determined using a murine fibroblast cell line L929 (acquired from the American Type Culture Collection (ATCC)), according to the International Organization for Standardization (ISO) [25]. Figure 6 shows that all formulations at different concentrations had high levels of cytocompatibility, confirming that they are viable, safe structures without any notable cell death. In fact, for many of the nanocomposite concentrations tested, fibroblast viability actually increased, compared to the controls.

**Figure 7:**
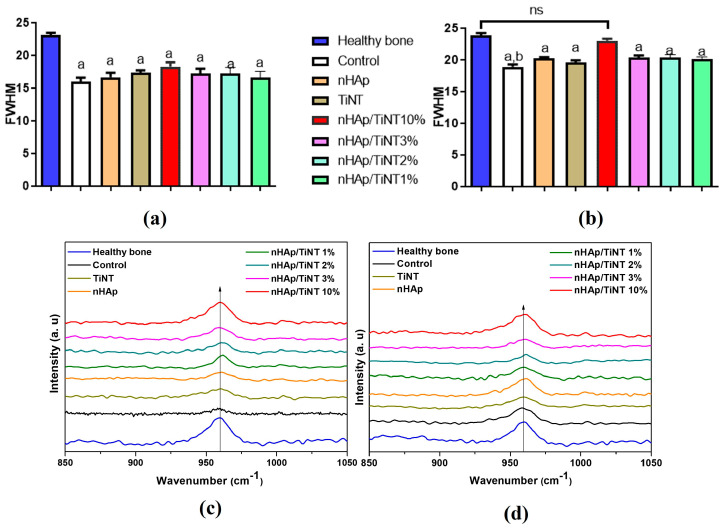
Statical analyses of bone growth at 15 days (**a**) and 30 days (**b**) and at 15 days (**c**) and (**d**) 30 days on nanocomposites after implantation in rat tibias. Raman spectra showing the peak ≈ 960 cm^−1^ of the treated and untreated experimental groups compared to healthy bone. a = *p* < 0.0001 when compared to the healthy bone group; b = *p* < 0.0001 when compared to the nHAp/TiNT10 % group; ns = not significant.

Statistical analysis of the rat tibias with bone defects from all groups in the two experimental periods of 15 and 30 days (Figure 7) were performed through Raman spectroscopy, using the phosphate peak ~960 cm^−1^ (this investigated peak is characteristic of the hydroxyapatite), which was used as a marker of bone repair [27,32].

The peak at ~960 cm^−1^ (ν_1_ ${\mathrm{PO}}_{4}^{3-}$) was used to fit the integrated area for each group, the spectra were averaged, and then the full width at half height (FWHM) was calculated. The spectra (Figure 7c,d) showed that the groups have the same peak positions, but with different intensities and widths. nHAp/TiNT10% (Figure 7c) showed a higher peak intensity compared to the other groups evaluated. The Raman band for the healthy group (bone) was used as a reference. This area is strictly linked to the amount of hydroxyapatite in the newly formed bone, that is, the greater the intensity is, the greater the concentration of phosphate is [32].

Statistical analysis of phosphate peak data (960 cm^−1^) was performed (Figure 7a,b). After 15 days (Figure 7a), the results did not indicate statistical differences between the groups tested in relationship to the healthy bone group, that is, there was no bone repair among the evaluated groups; however, there was a tendency for higher values in the nHAp/TiNT10% group. After 30 days (Figure 7b), all groups showed the same statistical behavior observed over the 15-day period, except for the nHAp/TiNT10% group, which showed statistical differences in the area, compared to the control group (without any treatment). Regarding the healthy group (bone), the nHAp/TiNT10% group did not show significant differences, showing that the two groups are compatible and that, therefore, there was bone repair for the nHAp/TiNT10% group.

**Figure 8:**
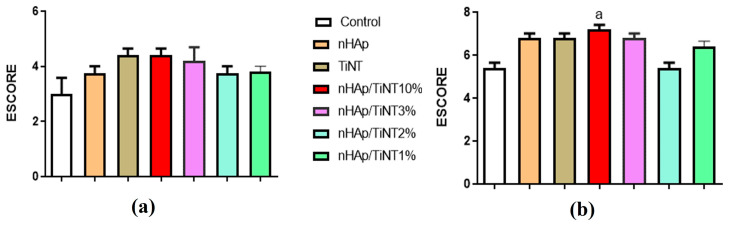
Histopathological analysis of bone repair for the two experimental periods, 15 days (**a**) and 30 days (**b**), for nHAp, TiNT, nHAp/TiNT1%, nHAp/TiNT2%, nHAp/TiNT3%, and nHAp/TiNT10%. a = *p* <0. 0001 when compared to the control group without any treatment.

Semiquantitative histopathological analyses (Figure 8a,b) were performed to evaluate bone repair for the two experimental periods, after 15 and 30 days. The bone repair process was evaluated according to the scores mentioned in the methodology of Section 2.5. For the experimental period of 15 days, the histological analysis showed bone neoformation from the edges and inside the defect, consisting of connective tissue areas with bone differentiation, as well as immature bone trabeculae originating from the edges of the lesion and growing toward the center. An attempt to close the bone defect was visualized, though it was not sufficient to fill the entire area in most groups. Despite the variation identified between the different groups of the nanocomposites, nHAp/TiNT1%, nHAp/TiNT2%, nHAp/TiNT3%, and nHAp/TiNT10%, and the other TiNT and nHAp groups, there was no statistically significant difference (Figure 8a).

After 30 days, bone neoformation was observed for the entire extension of the defects, consisting of trabeculae composed of mature bone tissue or in an advanced stage of bone organization, with variable thickness, from one-third corresponding to the adjacent original cortical bone until completely filled, which was similar in thickness to the adjacent cortical bone. Significant differences were found between the nHAp/TiNT10% groups and the control (without any treatment) (Figure 8b). Bone repair was statistically higher in the nHAp/TiNT10% group (*p* < 0.0100), compared to the control. Comparing the statistical data of the histology of all groups with the treatment, it appears that there were no significant differences between them (nHAp/TiNT1%, nHAp/TiNT2%, nHAp/TiNT3%, nHAp/TiNT10%, TiNT, and nHAp). So far, there has been no investigation into the in vivo performance of nHAp/TiNTs nanocomposites at different percentages of TiNTs (1, 2, 3, and 10% by weight), such as for the nanocomposites produced in this work for bone regeneration. In addition, this same study demonstrated that all the nanocomposites after 30 days showed bone regeneration. After 15 days, a trend toward better repair can be observed for the nHAp/TiNT10% and TiNT groups, which was confirmed for the 30-day experimental group.

**Figure 9:**
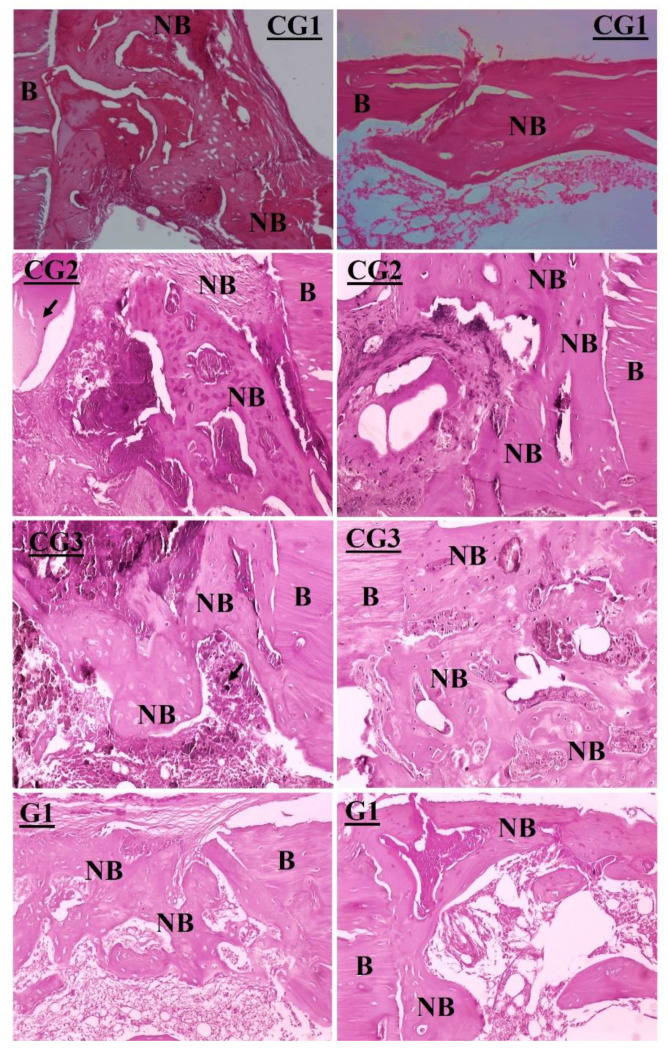

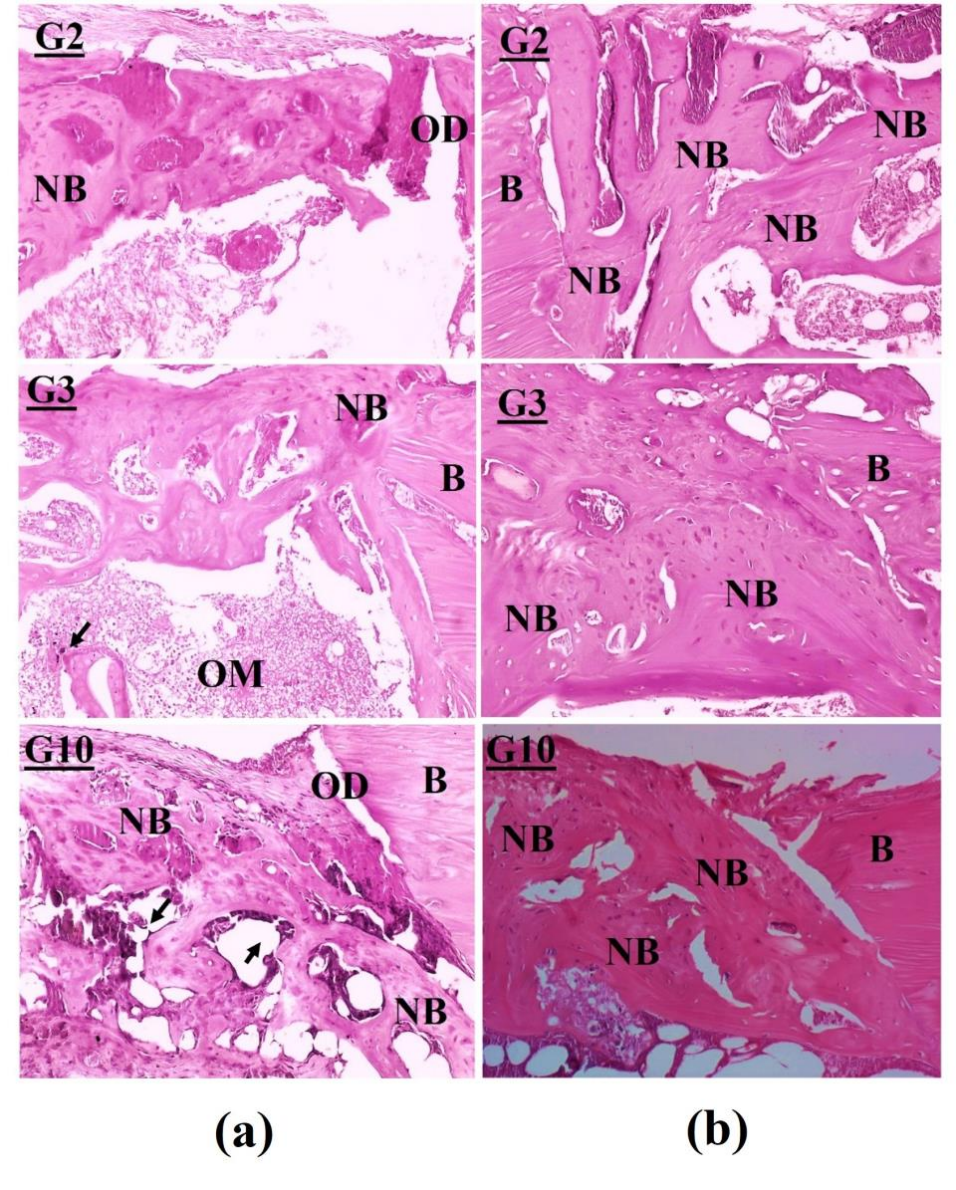
Histological analysis of the groups of nanocomposites CG1 (control group without treatment), CG2 (nHAp), CG3 (TiNT), G1 (nHAp/TiNT1%), G2 (nHAp/TiNT2%), G3 (nHAp/TiNT3%), and G10 (nHAp/TiNT10%) on the left after 15 days (**a**); images on the right after 30 days (**b**). Bone marrow (OM), arrow (presence of material, foreign body), newly formed bone (NB), original bone (B), and bone defect (OD).

In Figure 9 (the left side shows bone repair in the control and experimental groups after 15 days), neo-tissue formation consisting of connective tissue and bone differentiation, as well as immature bone trabeculae originating from the edges of the lesion and growing toward the center of the defects, was observed when the nanocomposites were used (Figure 9; right side of the image shows the bone repair in the control and experimental groups after 30 days). It is also possible to observe a large number of nanocomposites in the bone matrix, as indicated by the arrows.

After 30 days, it can be seen that bone neoformation in the defects was quite evident, with a greater number of trabeculae of mature bone tissue and a total filling of the defect and variation only in the thickness of the filling when compared to the adjacent cortical bone, as classified by histological scores based on the different parameters in the bone healing process. The presence of islands of connective tissue, rich in blood capillaries, inserted into the defect was also seen (Figure 9; right side of the image shows bone repair in the control and experimental groups for the 30-day period). The presence of nanocomposites in the bone matrix, as indicated by arrows, was also noticed. Necrosis was not observed in any of the implantable nanocomposite groups.

## 4. Conclusions

Here, we report, for the first time, the synthesis of nHAp/TiNT nanocomposites at different concentrations (1%, 2%, 3%, and 10% by weight of TiNT), showing promising characteristics for orthopedic applications. The crystalline nHAp structure was the main characteristic and stable phase of the nanocomposite. Furthermore, Rietveld refinement confirmed the structure of nHAp, and the addition of TiNT affected the morphology of the nanocomposites, decreasing the average crystallite size from 54 nm (nHAp) to 34 nm (nHAp/TiNT10%). Cytotoxic activity using a fibroblast cell lineage at different concentrations revealed that there was no notable cell death at any of the concentrations tested. More importantly, an in vivo study performed using rats with osteoporosis clearly showed that all nanocomposites accelerated bone regeneration and that the nHAp/TiNT10% group statistically increased bone neoformation after 30 days of implantation, compared to the control group without any treatment, as demonstrated by Raman spectroscopy. In summary, this study introduces new significantly promising nanocomposites composed of nHAp and TiNT for numerous orthopedic applications.

## Figures and Tables

**Table 1:** Results of the Rietveld refinement of lattice parameters (a, b, and c = axial lengths; α, β, and γ = angles, unit cell volume (V), and cell density (ρ), respectively, for the nHAp sample).

Lattice Parameters (Hydroxyapatite)					
a = b (Å)	c (Å)	α = β (◦)	g (◦)	V (Å^3^)	Ρ (g/cm^3^)
9.436(1)	6.8875(7)	90	120	531.1 (1)	3.141

**Table 2:** Crystallinity index estimated by Raman spectroscopy and XRD.

Samples	CI_Raman_ (Average)	CI_X-ray_ (Average)
nHAp	0.71	0.42
nHAp/TiNT1%	0.62	0.20
nHAp/TiNT2%	0.63	0.22
nHAp/TiNT3%	0.50	0.19
nHAp/TiNT10%	0.49	0.18

**Table 3:** nHAp/TiNT nanocomposites (1%, 2%, 3%, and 10%) at different concentrations showing crystallite size.

Samples	2θ [°] Peak (002)	(2θ) FWHM	Crystallite Size XRD (nm)
nHAp	25.87	0.208	54
nHAp/TiNT1%	25.88	0.270	37
nHAp/TiNT2%	25.89	0.225	48
nHAp/TiNT3%	25.88	0.273	36
nHAp/TiNT10%	25.89	0.286	34

**Table 4:** Zeta potential, dynamic light scattering, and polydispersion index (PdI) measurements of the materials of interest to this study.

Samples	Zeta Potential (mV)	Hydrodynamic Diameter (nm)	PdI
TiNT	−27.40	300.4	0.552
nHAp	−5.160	4888	0.274
nHap/TiNT1%	−0.921	3824	0.266
nHap/TiNT2%	−0.391	3416	0.215
nHap/TiNT3%	−0.019	3606	0.363
nHap/TiNT10%	−1.520	2247	0.586

**Table 5:** The presence of Ti in the nanocomposites and the Ca/P atomic ratios.

Samples	Ti %	Ca/P %
nHAp	-	1.93
nHAp/TiNT1%	0.37	1.92
nHAp/TiNT2%	0.40	2.13
nHAp/TiNT3%	0.61	3.03
nHAp/TiNT10%	8.53	2.08

## Data Availability

Data supporting the reported results can be requested by e-mail.

## Supplementary Materials

The following supporting information can be downloaded at: https://www.mdpi.com/article/10.3390/jfb13040306/s1, Figure S1: Transmission electron microscopy images showing the nanometric diameter (~10nm) of titanate nanotubes; Figure S2: Images of the tibias of the experimental groups after 30 days of implantation of the nanobiomaterials: nHAp, nHAp/TiNT1%, nHAp/TiNT2%, nHAp/TiNT3% e nHAp/TiNT10%.

## References

[1] Armiento A.R., Hatt L.P., Rosenberg G.S., Thompson K., Stoddart M.J. (2020). Functional Biomaterials for Bone Regeneration: A Lesson in Complex Biology. Adv. Funct. Mater..

[2] Qu H., Fu H., Han Z., Sun Y. (2019). Biomaterials for Bone Tissue Engineering Scaffolds: A Review. RSC Adv..

[3] Nuss K.M.R., von Rechenberg B. (2008). Biocompatibility Issues with Modern Implants in Bone—A Review for Clinical Orthopedics. Open Orthop. J..

[4] Tavoni M., Dapporto M., Tampieri A., Sprio S. (2021). Bioactive Calcium Phosphate-Based Composites for Bone Regeneration. J. Compos. Sci..

[5] Lahiri D., Ghosh S., Agarwal A. (2012). Carbon Nanotube Reinforced Hydroxyapatite Composite for Orthopedic Application: A Review. Mater. Sci. Eng. C.

[6] Balani K., Chen Y., Harimkar S.P., Dahotre N.B., Agarwal A. (2007). Tribological Behavior of Plasma-Sprayed Carbon Nanotube-Reinforced Hydroxyapatite Coating in Physiological Solution. Acta Biomater..

[7] Barua S., Yoo J.-W., Kolhar P., Wakankar A., Gokarn Y.R., Mitragotri S. (2013). Particle shape enhances specificity of antibody-displaying nanoparticles. Proc. Natl. Acad. Sci. USA.

[8] Zanin H., Rosa C.M.R., Eliaz N., May P.W., Marciano F.R., Lobo A.O. (2015). Assisted Deposition of Nano-Hydroxyapatite onto Exfoliated Carbon Nanotube Oxide Scaffolds. Nanoscale.

[9] Long M., Rack H.J. (1994). Titanium Alloys in Total Joint Replacement—a Materials Science Perspective Marc. Appl. Cogn. Psychol..

[10] Niinomi M. (2007). Recent Research and Development in Metallic Materials for Biomedical, Dental and Healthcare Products Applications. Mater. Sci. Forum.

[11] Liu X., Chu P.K., Ding C. (2004). Surface modification of titanium, titanium alloys, and related materials for biomedical applications. Mater. Sci. Eng. R Rep..

[12] Li J., Cui X., Hooper G.J., Lim K.S., Woodfield T.B.F. (2020). Rational Design, Bio-Functionalization and Biological Performance of Hybrid Additive Manufactured Titanium Implants for Orthopaedic Applications: A Review. J. Mech. Behav. Biomed. Mater..

[13] Wang Y., Zhang D., Wen C., Li Y. (2015). Processing and Characterization of SrTiO_3_–TiO_2_Nanoparticle–Nanotube Heterostructures on Titanium for Biomedical Applications. ACS Appl. Mater. Interfaces.

[14] Zazakowny K., Lewandowska-Łańcucka J., Mastalska-Popławska J., Kamiński K., Kusior A., Radecka M., Nowakowska M. (2016). Biopolymeric hydrogels—nanostructured TiO2 hybrid materials as potential injectable scaffolds for bone regeneration. Colloids Surf. B Biointerfaces.

[15] Sallem F., Boudon J., Heintz O., Séverin I., Megriche A., Millot N. (2017). Synthesis and Characterization of Chitosan-Coated Titanate Nanotubes: Towards a New Safe Nanocarrier. Dalt. Trans..

[16] Kasuga T., Hiramatsu M., Hoson A., Sekino T., Niihara K. (1998). Formation of titanium oxide nanotube. Langmuir.

[17] Wang L., Liu W., Wang T., Ni J. (2013). Highly Efficient Adsorption of Cr(VI) from Aqueous Solutions by Amino-Functionalized Titanate Nanotubes. Chem. Eng. J..

[18] Marques T.M., Sales D.A., Silva L.S., Bezerra R.D., Silva M.S., Osajima J.A., Ferreira O.P., Ghosh A., Filho E.C.S., Viana B.C. (2020). Amino-functionalized titanate nanotubes for highly efficient removal of anionic dye from aqueous solution. Appl. Surf. Sci..

[19] Barbosa M.C., Messmer N.R., Brazil T.R., Marciano F.R., Lobo A.O. (2013). The Effect of Ultrasonic Irradiation on the Crystallinity of Nano-Hydroxyapatite Produced via the Wet Chemical Method. Mater. Sci. Eng. C.

[20] Oliveira F.C., Carvalho J.O., Gusmão S.B.S., Gonçalves L.D.S., Mendes L.M.S., Freitas S.A.P., Gusmão G., Viana B.C., Marciano F.R., Lobo A.O. (2019). High loads of nano-hydroxyapatite/graphene nanoribbon composites guided bone regeneration using an osteoporotic animal model. Int. J. Nanomed..

[21] Renaudin G., Laquerrière P., Filinchuk Y., Jallot E., Nedelec J.M. (2008). Structural Characterization of Sol-Gel Derived Sr-Substituted Calcium Phosphates with Anti-Osteoporotic and Anti-Inflammatory Properties. J. Mater. Chem..

[22] Larson A.C., Von Dreele R.B. (2004). General Structure Analysis System (GSAS). Alamos Natl. Lab. LAUR.

[23] Poralan G.M., Gambe J.E., Alcantara E.M., Vequizo R.M. (2015). X-ray diffraction and infrared spectroscopy analyses on the crystallinity of engineered biological hydroxyapatite for medical application. IOP Conf. Ser. Mater. Sci. Eng..

[24] Czekanska E.M. (2011). Assessment of Cell Proliferation with Resazurin-Based Fluorescent Dye. Methods Mol. Biol..

[25] (2009). Biological Evaluation of Medical Devices—Part 5: Tests for in Vitro Cytotoxicity.

[26] Alves A.M.M., Fortaleza L.M.D.M., Filho A.L.M.M., Ferreira D.C.L., da Costa C.L.S., Viana V.G.F., Santos J.Z.L.V., de Oliveira R.A., Gusmão G.O.D.M., Soares L.E.S. (2018). Evaluation of bone repair after application of a norbixin membrane scaffold with and without laser photobiomodulation (λ 780 nm). Lasers Med. Sci..

[27] Monzem S., Sônego D.A., Martini A.D.C., Moura A.P.B.D., da Silva F.G., de Faria J.L.B., de Souza R.L. (2018). Raman spectroscopic of osteoporosis model in mouse tibia in vivo. Vib. Spectrosc..

[28] Hedner E., Linde A. (1995). Efficacy of Bone Morphogenetic Protein (BMP) with Osteopromotive Membranes—an Experimental Study in Rat Mandibular Defects. Eur. J. Oral Sci..

[29] Gusmão S.B., Ghosh A., Marques T.M., Vieira L.H.S., Ferreira O.P., Silva-Filho E.C., Lobo A.O., Osajima J.A., Viana B.C. (2019). Titanate-based one-dimensional nano-heterostructure: Study of hydrothermal reaction parameters for improved photocatalytic application. Solid State Sci..

[30] Vieira E.G., Sousa P.A.A., Matos J.M.E., Santos M.R.M.C., Ininga B. (2013). Síntese Pelo Método Da Coprecipitação e Caracterização Estrutural Do Tungstato de Cálcio Com Estrutura Tipo Scheelita (Synthesis by the Coprecipitation Method and Structural Characterization of Calcium Tungstate with Scheelite Type Structure). Cerâmica.

[31] Cavalcante L.S., Longo V.M., Sczancoski J.C., Almeida M.A.P., Batista A.A., Varela J.A., Orlandi M.O., Longo E., Li M.S. (2012). Electronic Structure, Growth Mechanism and Photoluminescence of CaWO 4 Crystals. CrystEngComm.

[32] Vieira E., Silva M., Maia-Filho A., Ferreira D., Figuerêdo-Silva J., Rovaris K., Fialho A., Leite-Oliveira A., de Oliveira A.M., da Fonseca M. (2021). Effect of Cerium-Containing Hydroxyapatite in Bone Repair in Female Rats with Osteoporosis Induced by Ovariectomy. Minerals.

[33] Cho J.S., Um S.-H., Yoo D.S., Chung Y.-C., Chung S.H., Lee J.-C., Rhee S.-H. (2013). Enhanced osteoconductivity of sodium-substituted hydroxyapatite by system instability. J. Biomed. Mater. Res. Part B Appl. Biomater..

[34] Viana B.C., Ferreira O.P., Filho A.G.S., Hidalgo A.A., Filho J.M., Alves O.L. (2011). Highlighting the mechanisms of the titanate nanotubes to titanate nanoribbons transformation. J. Nanoparticle Res..

[35] Rodrigues B.V.M., Leite N.C.S., das Neves Cavalcanti B., da Silva N.S., Marciano F.R., Corat E.J., Webster T.J., Lobo A.O. (2016). Graphene Oxide/Multi-Walled Carbon Nanotubes as Nanofeatured Scaffolds for the Assisted Deposition of Nanohydroxyapatite: Characterization and Biological Evaluation. Int. J. Nanomed..

[36] Medeiros J.S., Oliveira A.M., De Carvalho J.O., Ricci R., Martins M.D.C.D.C.E., Rodrigues B.V.M., Webster T.J., Viana B.C., Vasconcellos L.M.R., Canevari R. (2018). Nanohydroxyapatite/Graphene Nanoribbons Nanocomposites Induce In Vitro Osteogenesis and Promote in Vivo Bone Neoformation. ACS Biomater. Sci. Eng..

[37] Ferreira O.P., Filho A.G.S., Filho J.M., Alves O.L. (2006). Unveiling the structure and composition of titanium oxide nanotubes through ion exchange chemical reactions and thermal decomposition processes. J. Braz. Chem. Soc..

[38] Bertrand N., Grenier P., Mahmoudi M., Lima E.M., Appel E.A., Dormont F., Lim J.-M., Karnik R., Langer R., Farokhzad O.C. (2017). Mechanistic understanding of in vivo protein corona formation on polymeric nanoparticles and impact on pharmacokinetics. Nat. Commun..

[39] Casals E., Galán A.M., Escolar G., Gallardo M., Estelrich J. (2003). Physical Stability of Liposomes Bearing Hemostatic Activity. Chem. Phys. Lipids.

