# Supplementary material for: Nanohydroxyapatite/Titanate Nanotube Composites for Bone Tissue Regeneration
Source: J Funct Biomater. 2022 Dec 17;13(4):306. doi: 10.3390/jfb13040306 (PMC9786793; doi:10.3390/jfb13040306)
Supplement: Supplementary file 1 [file jfb-13-00306-s001.zip › jfb-2036850-supplementary.pdf]

Supplementary Information

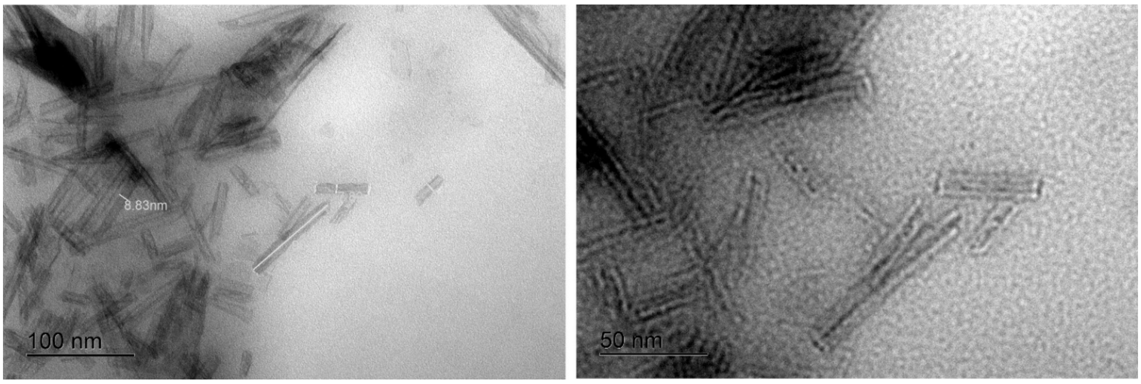

Figure S1. Transmission electron microscopy images showing the nanometric diameter (~10nm) of titanate nanotubes.

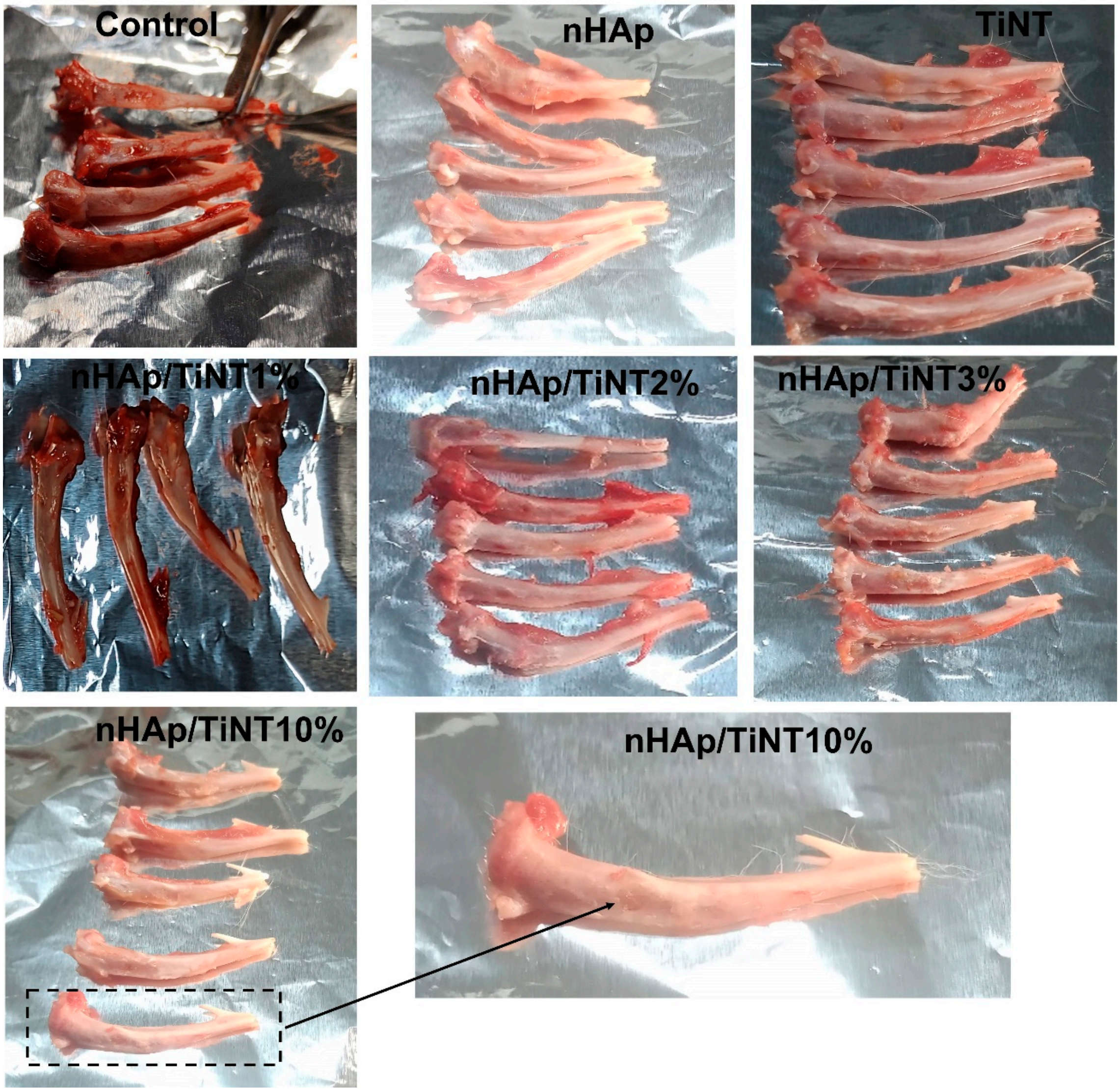

Figure S2: Images of the tibias of the experimental groups after 30 days of implantation of the nanobiomaterials: nHAp, nHAp/TiNT1%, nHAp/TiNT2%, nHAp/TiNT3% e nHAp/TiNT10%.
